# Pulmonary Complications in Children Following Hematopoietic Cell Transplantation: A Case Report and Review of the Diagnostic Approach

**DOI:** 10.3389/fonc.2021.772411

**Published:** 2021-11-08

**Authors:** Lama Elbahlawan, Jenny McArthur, Cara E. Morin, Hafeez Abdelhafeez, M. Beth McCarville, Robert E. Ruiz, Saumini Srinivasan, Amr Qudeimat

**Affiliations:** ^1^Division of Critical Care Medicine, St. Jude Children’s Research Hospital, Memphis, TN, United States; ^2^Department of Diagnostic Imaging, St. Jude Children’s Research Hospital, Memphis, TN, United States; ^3^Department of Surgery, St. Jude Children’s Research Hospital, Memphis, TN, United States; ^4^Department of Pathology, St. Jude Children’s Research Hospital, Memphis, TN, United States; ^5^Division of Pulmonary, University of TN Health Science Center (UTHSC), Memphis, TN, United States; ^6^Department of Bone Marrow Transplant and Cellular Therapy, St. Jude Children’s Research Hospital, Memphis, TN, United States

**Keywords:** pulmonary complications, hematopoietic (Stem) cell transplantation (HCT), lung biopsy, broncho alveolar lavage (BAL), diagnostic approach, diagnostic imaging

## Abstract

Pulmonary complications are common in children following hematopoietic cell transplantation (HCT) and contribute to their morbidity and mortality. Early diagnosis is essential for management and prevention of progression of lung injury and damage. In many cases, diagnosis can be challenging and may require diagnostic imaging and more invasive testing such as bronchoscopy and lung biopsy. We report the case of a 12-year-old girl who developed recurrent episodes of acute respiratory failure requiring intensive care unit admission in the post-HCT phase and describe the diagnostic and multidisciplinary approach for her management. In addition, we review the diagnostic approach of pulmonary complications post-HCT and highlight the utility and risks of bronchoscopy and lung biopsy in these children.

## Introduction

Pulmonary complications are common in children following hematopoietic cell transplantation (HCT) and contribute to morbidity and mortality in the post-HCT phase (1–3). Early identification, diagnosis, and treatment of these complications are essential to limit morbidity and improve outcome. However, diagnosis can be quite challenging as etiologies can overlap and obscure diagnosis. In such complicated cases, laboratory testing and diagnostic imaging may not establish diagnosis and further invasive diagnostic testing such as bronchoscopy and lung biopsy might be needed. We report a 12-year-old girl who developed recurrent episodes of respiratory distress requiring intensive care unit (ICU) admission in the post-HCT phase and describe the diagnostic challenges and the multidisciplinary approach that is crucial in similar situations. Furthermore, we present a review of the diagnostic approach of pulmonary complications post-HCT and highlight the utility of bronchoscopy and lung biopsy to establish diagnosis in these children.

## Case Report

**Figure 1:**
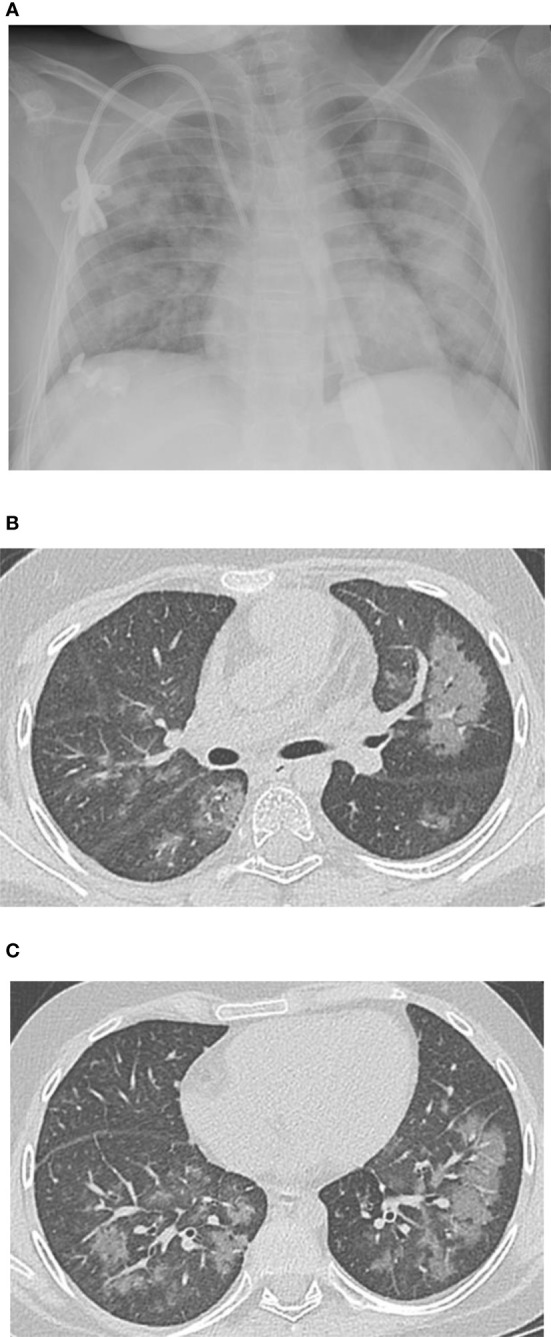
**(A)** Chest x-ray obtained Day +42 demonstrates diffuse airspace disease bilaterally with more focal areas of consolidation in the left lung. **(B, C)** Thin-section computed tomography (CT) obtained Day +49 (from 2^nd^ transplant) demonstrate diffuse ground-glass opacification involving all 5 lobes.

**Figure 2:**
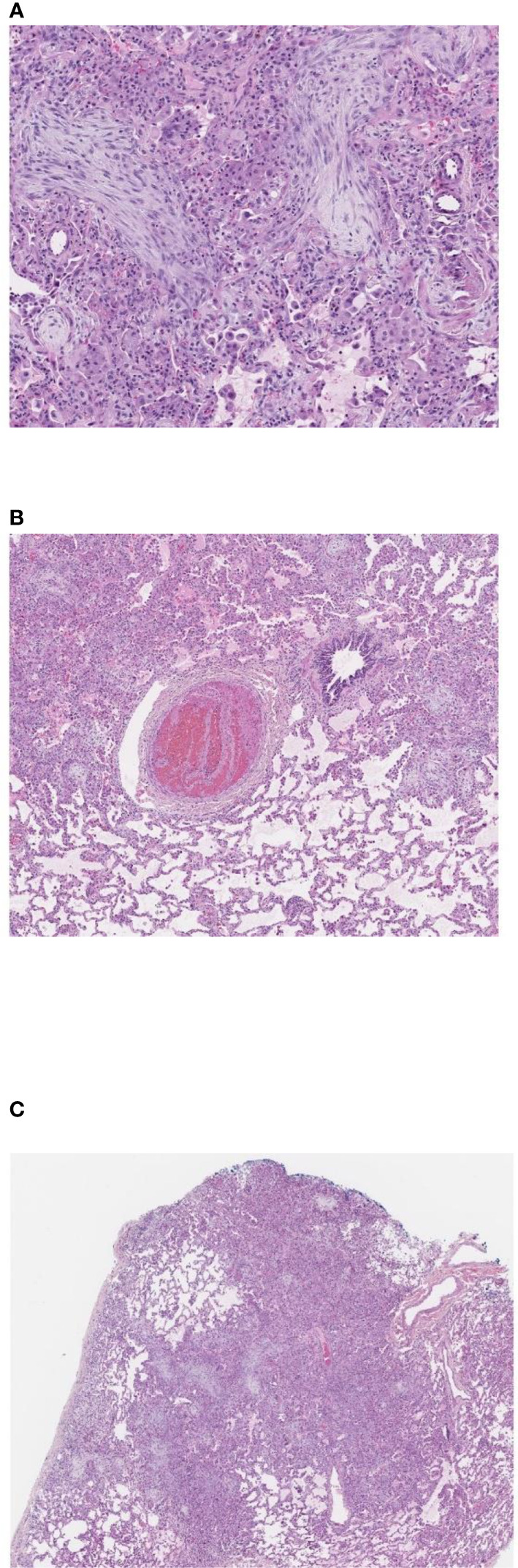
Pulmonary wedge biopsy, Day +55. **(A)** Patchy consolidation, 2x. **(B)** Organizing pneumonia with fibroblastic proliferation in terminal airways and numerous macrophages occupying airspaces, 10x. **(C)** Pulmonary arterial thrombus with alternating bands of fibrin and platelets (lines of Zahn), 4x. **(A–C)**. Hematoxylin and eosin stain, magnifications using Leica Biosystems Aperio ImageScope.

**Figure 3:**
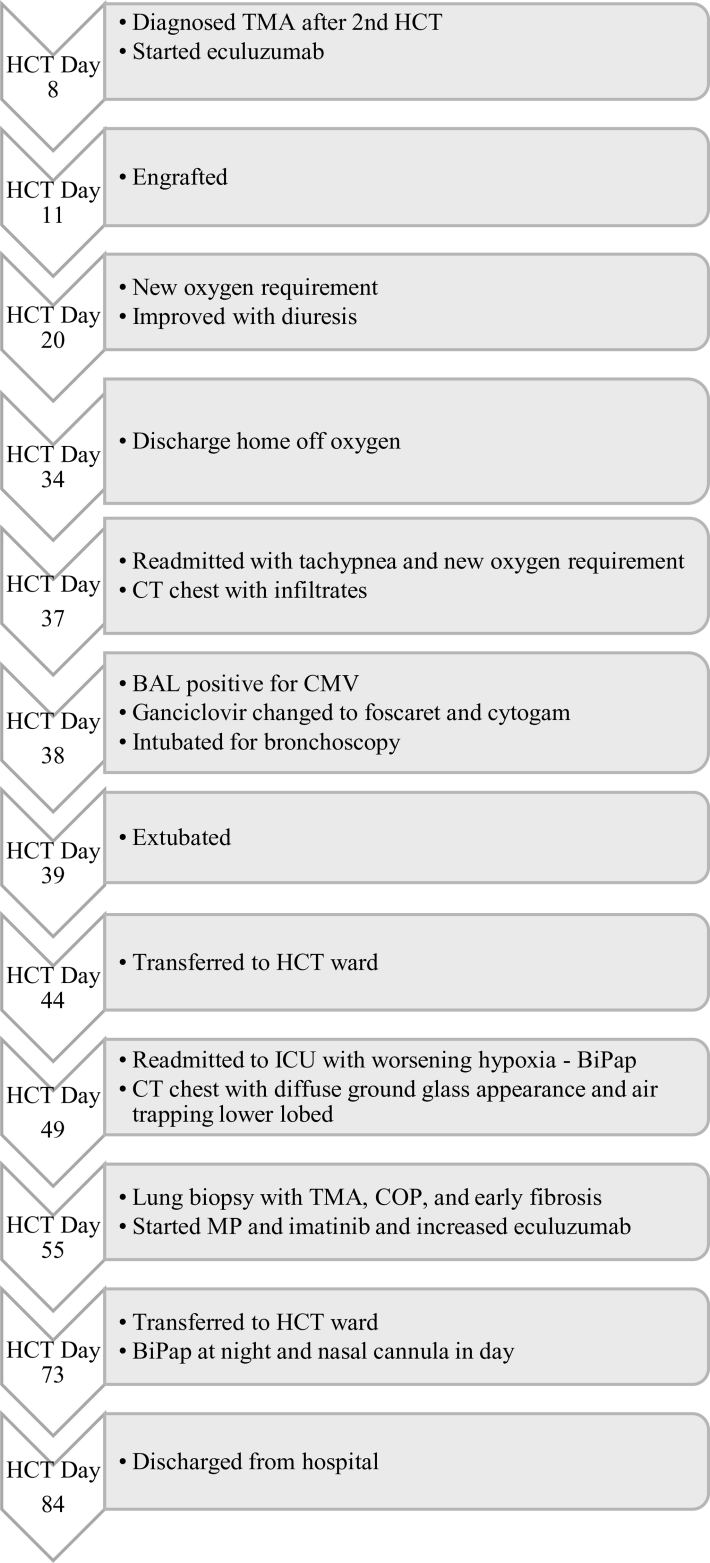
Timeline of events for ICU admission.

A 12-year-old female patient with a history of T-cell acute lymphoblastic leukemia presented to our ICU on Day +38 after her second haploidentical HCT due to a failed extubation in the OR post bronchoscopy. Our patient had previously undergone a haploidentical HCT with prep regimen consisting of fludarabine, thiotepa, melphalan, cyclophosphamide and rabbit ATG followed by a TCRα/β+ and CD19+ depleted graft infusion. GCSF was administered until engraftment and CD45RA-depleted donor lymphocyte infusion was given post engraftment for immune reconstitution. Our patient’s first haploidentical HCT was complicated by Cytomegalovirus (CMV) viremia and acute grade II gastrointestinal graft versus host disease (GVHD). CMV reactivation was treated with ganciclovir, foscarnet and cytogam administration and GVHD became quiescent with oral budesonide and beclomethasone. She had a CNS relapse on Day +150, with subsequent bone marrow relapse. She underwent remission induction chemotherapy and proceeded to have a second haploidentical HCT. During her second HCT, she received reduced intensity conditioning with fludarabine, thiotepa, melphalan, cyclophosphamide, and rabbit ATG, followed by a TCRα/β+ depleted graft and subsequent memory T cell infusion (CD45A+ depletion). Our patient’s second HCT was complicated by CMV reactivation requiring ganciclovir therapy as well as rising LDH, complement level (CH50), proteinuria, and acute kidney injury suspicious for transplant-associated thrombotic microangiopathy (TMA). She was started on eculizumab on Day +8 after her second HCT and achieved engraftment on Day +11. Engraftment was complicated by fevers and hyperferritinemia suspicious for secondary hemophagocytic lymphohistiocytosis treated briefly with Anakinra that was discontinued since she did not meet criteria for diagnosis of hemophagocytic lymphohistiocytosis. She was switched from sirolimus to ruxolitinib for GVHD prophylaxis. She developed a brief oxygen requirement on Day +20, which responded well to diuresis and she was discharged to home on Day +34. On Day +37 she was readmitted with tachypnea, a new oxygen requirement, and a diffuse bilateral airspace disease on chest x-ray (**Figure 1A**). A bronchoscopy was performed on day +38 to evaluate for an infectious etiology after elective intubation, and she was successfully extubated on Day +39. Broncho-alveolar lavage fluid was PCR positive for CMV, but no other infectious source was identified. Her ganciclovir was switched to foscarnet with cytogam added to improve coverage. Her condition improved and she transferred back to the HCT ward on Day +44. On Day +49, she was readmitted to the ICU with worsening hypoxia. CT scan showed diffuse ground glass appearance with air trapping in the lower lobes (**Figures 1B, C**). Despite serum CMV titers remaining low, her respiratory status continued to worsen. Lung biopsy by video-assisted thoracoscopic surgery (VATS) was performed on Day +55 due to uncertainty surrounding her diagnosis. Her lung biopsy demonstrated organizing pneumonia with patchy consolidation (**Figure 2A**), characterized by prominent fibroblastic proliferation in terminal airways and numerous macrophages occupying airspaces, compatible with cryptogenic organizing pneumonia (COP) (**Figure 2B**). An occlusive arterial thrombus was noted (**Figure 2C**), a possible sequela of TMA. Given these biopsy findings, she was started on methylprednisolone 1 mg/kg/day for COP. Steroids were well tolerated and were tapered over a period of 4 months. Her eculizumab dosing was increased to twice per week for better control of her TMA, and imatinib was added to prevent progression of fibrosis. Her respiratory status slowly improved, and she was transitioned back to the HCT unit on Day +73, with stable settings on bilevel positive airway pressure (BIPAP) support at night and minimal (1–2 liters/min) supplemental nasal cannula oxygen during the day. She was discharged to home on Day +84 with the same level of respiratory support. Imatinib was discontinued on day +169 and she was continued on Ruxolitinib. Clinical assessment on her last follow-up on day +315 showed continued improvement in her lung function with tolerance of her gradual wean of the BIPAP support. Her clinical course is summarized in **Figure 3**.

## Pulmonary Complications Post-HCT

Pulmonary complications post-HCT can be related to infectious or non-infectious etiologies. Infections causing pneumonia or ARDS can occur at any stage during the HCT course. However, infectious organisms will vary with the specific immune deficiency, whether humoral or cellular, depending on the timing of transplant. The rate of infectious complications has decreased with better strategies for pre-emptive testing and surveillance as well as use of antimicrobial prophylaxis. However, the incidence of non-infectious lung injury is still on the rise. Late onset non-infectious pulmonary complications usually follow a more predictable timeline after HCT (**Table 1**) (1, 4–9).

**Table 1:** Non-infectious pulmonary complications post-HCT.

Pulmonary complication	Onset post- HCT	Presentation	Characteristic findings	Histopathology	Treatment
IPS	Within 120 d	Cough, hypoxia, dyspnea	Widespread alveolar injury	Diffuse alveolar damage, interstitial pneumonitis	Corticosteroids TNF inhibitors
PERDS	Within 5 –7 d of neutrophil engraftment	Fever, rash, hypoxia	Non-cardiogenic pulmonary edema		Fluid overload prevention, diuretics, corticosteroids
DAH	Within 100 d	Cough, progressive hypoxia	Progressive bloody BAL	>20% hemosiderin-laden macrophages	Platelet count >50,000, correct coagulopathy, corticosteroids, inhaled TXA/inhaled rFVII
BOS	3–24 m	Cough, dyspnea, wheezing	Obstructive lung disease: FEV1/FVC<0.7 FEV1<75%	Intraluminal fibrotic plug of granulation tissue within bronchioles, spare alveoli and alveolar ducts	Corticosteroids, inhaled corticosteroids. azithromycin, and montelukast
COP	2–12 m	Cough, dyspnea, fever, crackles	Restrictive lung disease: reduced TLC and ↓DLCO	Granular plugs of bronchioles extending into alveoli with interstitial inflammation and fibrosis	Corticosteroids, macrolides
Pulmonary VOD		Dyspnea, pulmonary arterial hypertension with normal pulmonary occlusion pressure	Rare but fatal disease	Post-capillary pulmonary venular obstruction resulting in pulmonary vascular congestion	Usually not effective Corticosteroids Heparin Defibreotide
TA-TMA	Usually within 3 m	Microangiopathic hemolytic anemia, thrombocytopenia,proteinuria, hypertension	Schistocytes on blood smear ↑LDH ↑Blood sC5b-9	Endothelial injury with microthrombi, capillary flow obstruction due to fibrin-related aggregates, and platelet and leukocyte adhesion.	Cessation of offending agent Eculizumab Rituximab

IPS, idiopathic pneumonia syndrome; PERDS, peri-engraftment respiratory distress syndrome; DAH, diffuse alveolar hemorrhage; BOS, bronchiolitis obliterans; COP, cryptogenic organizing pneumonia; VOD, veno-occlusive disease; TA-TMA, transplant associated thrombotic microangiopathy; TNF, tumor necrosis factor; TXA, tranexamic acid; BAL, bronchoalveolar lavage; TLC, total lung capacity; FEV1, forced expiratory volume in one second; FVC, forced vital capacity; DLCO, diffusion lung capacity for carbon monoxide; d, day; m, months; ↑, increased.

## Diagnostic Approach

### Diagnostic Imaging

Pulmonary complications post-HCT are challenging for the radiologist due to pre-existing co-morbidities and co-existence of infectious and non-infectious etiologies, many of which have overlapping imaging findings. Chest radiographs are rarely sufficiently diagnostic and frequently falsely negative (10–17). Thus, chest computed tomography (CT) and specifically high-resolution chest CT has become commonplace in the management of pulmonary complications in post-HCT patients due to superior detection and characterization of parenchymal abnormalities (11–13, 17, 18). The term “high-resolution CT” engenders some confusion. Previously, this term referred to a technique of thin collimation (1–2 mm) coupled with a high-spatial-frequency reconstruction algorithm, performed by sampling the lung at staggered intervals (e.g., four slices evenly spaced from apices to bases or one slice every 2 cm) (19). Modern multi-detector helical CT scanners acquire “thin slices” (<1 mm), routinely allowing for contiguous imaging of the entire lung parenchyma and multiple high-resolution reconstructive algorithms to be performed following image acquisition. Imaging of the entire chest is typically performed without intravenous contrast during suspended full inspiration and repeated during suspended expiration. Examination of the lungs in expiration allows assessment of focal areas of air trapping that are not evident during inspiration. With modern-day multi-detector row CT scanners, the entire chest can be scanned in less than 1 sec for infant-sized patients and less than 2 sec for older children (20).

Imaging findings on CT must be interpreted within the appropriate clinical context. In the first 30 days, the frequency of infectious and non-infectious complications is similar (18). After 30 days, with immune system reconstitution, the frequency of infectious complications decreases and the spectrum of infectious organisms changes. In the neutropenic phase, infectious complications commonly include bacterial pneumonia, Respiratory syncytial virus (RSV), and invasive fungal pneumonia such as aspergillus. Bacterial pneumonia characteristically produces focal segmental or lobar pulmonary opacities. RSV and other viruses typically have a bilateral distribution and are often non-specific, ranging from normal CT scans to small nodules, ground-glass attenuation, and consolidation due to atelectasis or a combination of these (21, 22). Guidelines for imaging diagnosis of pulmonary aspergillosis include presence of one of the following patterns on CT: Dense, rounded lesion(s) with or without a halo sign (surrounding ground-glass hazy opacity); air crescent sign; cavity; or segmental lobar consolidation (23, 24). Criteria for other pulmonary mold diseases include prior criteria, with the addition of the reversed halo sign (central ground-glass opacity surrounded by denser crescentic consolidation). After 30 days, common pulmonary infectious agents include herpes simplex virus (HSV) or varicella zoster (VZ), CMV, pneumocystis jiroveci, and fungal pneumonia. As with RSV, findings with HSV, VZ, and CMV are non-specific and can include diffuse or multifocal areas of ground-glass attenuation, consolidations, and/or nodules (21, 25).

Non-infectious complications in the first 30 days include pulmonary edema, IPS, DAH, and PERDS. The most common complication in the first 30 days is pulmonary edema, which manifests on CT as enlargement of pulmonary vessels, diffuse ground glass opacification, septal thickening, and commonly cardiomegaly and/or pleural effusions. CT findings in IPS are non-specific and may include focal or diffuse airspace or reticular opacities in the setting of rapidly progressive respiratory failure. CT findings of DAH include diffuse ground-glass opacities and a “crazy-paving” pattern, related to intra- and inter-lobular thickening. Patients typically do not have cardiomegaly, prominent pulmonary vessels, or effusions differentiating this entity from edema. PERDS also demonstrates diffuse ground glass opacification, which can be associated with thickening of the interlobular septa, perihilar or peribronchial consolidation, and pleural effusions. Patients typically do not have cardiomegaly or other findings of pulmonary edema. Clinically, the presence of a skin rash and fever may be helpful for differentiation. In the early post-transplant period (30–100 days after HCT), IPS continues to be a common cause of respiratory symptoms. Additionally, acute GVHD can occur, although it is rare. CT findings of acute GVHD are non-specific and may include diffuse parenchymal opacities that resemble pulmonary edema (18).

Non-infectious late pulmonary complications (>100 days post HCT) include BOS, COP, or non-classifiable interstitial pneumonia (26). BOS demonstrates evidence of air trapping manifested as a mosaic attenuation pattern on CT. Mosaic attenuation is a finding of intermixed areas of low attenuation with areas of normal or increased attenuation. On expiratory images, the areas of decreased attenuation become more conspicuous. Thus, in patients >100 days post-HCT, it is recommended that CT be performed with inspiration and expiration phases (16, 27, 28). Additionally, patients with BOS demonstrate bronchiectasis. Findings of organizing pneumonia COP on CT include parenchymal consolidation with dilated bronchi, ground-glass opacities, nodular opacities, and/or the reversed-halo sign (18). Non-classifiable interstitial pneumonia is a third CT finding seen in post-HCT patients related to chronic GVHD. CT findings include ground glass opacities, reticulation, and the crazy-paving pattern in a predominant peribronchial distribution, as well as traction bronchiectasis progressing to more typical findings of fibrosis including bronchiectasis and honeycombing (18, 26).

## Pulmonary Function Test

The pulmonary function testing (PFT) is done routinely before HCT to assess baseline lung function. PFT does not usually have a role in early diagnosis of these pulmonary complications. However, PFT can be useful as a follow-up tool to assess response to therapy as well as a prognostic tool. BOS is characterized by an obstructive pattern with FEV1/FVC (forced expiratory volume in one second (FEV_1_)/forced vital capacity (FVC) ratio) <0.7 and FEV_1_ <75%. COP is characterized by a restrictive pattern with TLC less than the fifth percentile and normal FEV_1_/FVC (3).

## Bronchoscopy/Bronchoalveolar Lavage

Bronchoscopy with bronchoalveolar lavage (BAL) is usually performed with the acute onset and persistence of respiratory symptoms after antimicrobials initiation. In addition, if diffuse alveolar hemorrhage is highly suspected, BAL can confirm the diagnosis by retrieving sequential bloodier aliquots with BAL. Diagnostic yield of BAL in children post-HCT ranges from 30 to 67% and results in change of therapy in 34–68% of cases (**Table 2**). Viral and bacterial organisms are the most detected; the most common virus identified is CMV. Many factors can reduce the yield of this procedure, including duration of antimicrobial therapy before the procedure (30), acute GVHD GII-IV, and immunosuppressive therapy at the time of BAL (31). In a cohort of 57 post-HCT patients, shorter time between abnormal CXR and the BAL correlated with positive yield (2 days *vs* 6 days in patients with negative yield) (33). BAL yield is reported to be 2.5× higher when done within 4 days of presentation compared to later, and as high as 75% when performed within 24 h (38). Complications are reported in 1–31% of patients but are usually mild and short lived. Common complications include mild, transient hypoxia and bleeding. Recent introduction of metagenomic sequencing of these samples is promising and may increase the BAL yield by detecting a causative pathogen (39).

**Table 2:** Summary of studies evaluating BAL in children post-HCT with pulmonary complications.

Study	Patients (N)	Yield	Diagnosis	Complications
Ben-Ari et al (2001) (29)	52 Pediatrics	31% Therapy change: 34%	Bacterial: 22% Viral: 67% Fungal: 22%	1% Pulmonary hemorrhage
Eikenberry et al. (2005) (4)	90 Pediatrics	46%	Fungal: 15%	ND
Armenian et al. (2007) (30)	32 Pediatrics	50%	Viral>Fungal>Bacterial	Hemothorax: 3%
Kasow et al. (2007) (31)	89 Pediatrics	67%	Bacterial: 87% Viral: 23% Fungal: 11%	17% Hypoxia: 9%
Efrati et al. (2007) (32)	58(18 HCT) Pediatrics	53% Therapy change: 39%	Bacterial: 42% Viral: 36% Fungal: 6%	31% Transient hypoxia: 18% Mild bleeding: 6%
Forslow et al. (2010) (33)	57 Adult/Pediatrics	63% Therapy change: 47%	Bacterial: 24% Viral: 53% Fungal: 23%	Transient hypoxia: 9%
Qualter et al. (2014) (34)	65 Pediatrics	40%	Bacterial: 23% Viral: 14% Fungal: 12%	None
Nadimpalli et al. (2017) (35)	123 (75 HCT) Pediatrics	31% (HCT: 41%) Therapy change: 68%	Bacterial: 29% Viral: 28% Fungal: 36%	ND
Tang et al. (2018) (36)	130 Adult/Pediatrics	58% Therapy change: 61%	Bacterial: 38% Viral: 70% Fungal: 48%	ND
Eroglu-Ertugru et al. (2020) (37)	26 Pediatrics	54% Therapy change: 46%	Bacterial: 36% Viral: 50% Fungal: 21%	22% Mild/transient hypoxia: 20%

Includes studies with >10 patients from 2000 to 2020.

HCT, hematopoietic cell transplant; ND, not described.

## Lung Biopsy

### Surgical Lung Biopsy

Earlier reports of surgical lung biopsy (SLB) questioned the benefits of this procedure due to higher complication rates and lack of modification of therapy with the findings (40, 41). However, advances in surgical techniques as well as better understanding and therapies of pulmonary complications post-HCT improved yield and utility of this procedure. Surgical lung biopsy can be performed either as open biopsy through thoracotomy or currently more often thoracoscopically by video-assisted thoracoscopic surgery (VATS). Compared to other lung biopsy approaches, SLB usually provides better tissue samples, as tissue sampling is done under direct visualization. Recent reports show a relatively high diagnostic yield that ranges from 71 to 100% in children post-HCT (**Table 3**). In particular, lung biopsy is superior to BAL in diagnosing non-infectious etiologies such as COP and BOS. Infectious etiology is identified in 15–58% of pediatric HCT patients with this procedure. In fact, many infectious etiologies can be diagnosed and identified solely by the BAL without the need for lung biopsy, which may explain the lower infectious yield. In most instances (>70% of cases), lung biopsy leads to change in therapy and often involves adding immunosuppressive therapy. Children often require chest tube placement for a brief period post-operatively. Other uncommon complications that can occur in the context of lung biopsy include prolonged air leak, hemothorax, and splenic injury.

**Table 3:** Summary of studies evaluating lung biopsy in children post-HCT with pulmonary complications.

Study	Patients (N)	Type of LB	Yield	Diagnosis	Complications
Hayes-Jordan et al. (2002) (42)	19 Pediatrics		100% Therapy change: 89%	Infectious: 32% Non-infectious: 68%	Prolonged intubation: 37% Persistent Pneumothorax: 10%
Wang et al. (2004) (43)	35 Adult/Pediatrics		100% Therapy change: 63%	Infectious: 34% Non-infectious: 66%	8% Death: 3%
Gassas et al. (2013) (44)	48(59 LB) Pediatrics	OLB: 60% TT: 19% Percutanous: 21%	81% Therapy change: 42%	Infectious: 15% Non-infectious: 58%	No major complications
Qualter et al. (2014) (34)	16 (19 LB) Pediatrics	OLB: 16% VATS: 42% CT guided: 42%	94%	Infection: 58% Fibrosis: 21% GVHD: 16%	ChT: 42% Hemorrhage: 5%
Uhlving et al (2015) (45)	44 Adult/Pediatrics	OLB: 34 TBLB: 10	NA	BOS: 23 (52% fulfilled BOS criteria before biopsy) Other non-infectious: 21	Pneumothorax: 22% (only 1 patient required drainage) Infection at incision: 17.6% Minor bleeding: 2.9%
Ortega-Laureano et al. (2018) (46)	29 Pediatrics	OLB TT (76%)	96% Therapy change: 86%	COP: 27% Infectious: 24% DAH: 21%	ChT: 86% Bronchopleural fistula: 7% Splenic injury: 7% Hemothorax: 3%
					
Dieffenbach et al. (2019) (47)	48 Pediatrics	OLB TT	71% Therapy change: 79%	BOS/GVHD: 19% Other non-infectious: 17% Infectious: 23%	ChT>7d: 25% Hemothorax: 12.5% Splenic injury: 12.5%
Cleveland et al. (2020) (48)	68(50 HCT) Pediatrics	CT guided: 37% US guided: 56% Combined (CT+US): 7%	60%	Infectious: 81%	30% Major complications: 10% Pneumothorax/Hemoptysis/Death

Studies included having >10 patients from 2000 to 2020.

BOS, bronchiolitis obliterans; COP, cryptogenic organizing pneumonia; GVHD, graft versus host disease; ChT, chest tube; OLB, open lung biopsy; VATS, video-assisted thoracoscopic surgery; TBLB, transbronchial lung biopsy; TT, transthoracoscopic; US, ultrasound; CT, computed tomography.

### Percutaneous Lung Biopsy

Percutaneous lung biopsy is another approach to retrieve a lung tissue sample for examination. It can be performed by ultrasound if the lesion or abnormality is in the lung periphery or by CT guidance. Recently, Cleveland et al. reported the largest cohort of 68 immunocompromised pediatric patients (50 HCT) who underwent 73 percutaneous lung biopsies (48). Percutaneous lung biopsy was performed under either CT guidance (37%) or US guidance (56%) or both (7%), mostly for lesions that are pleural spaced (84%). Lesions were non-malignant in 48% of cases (total diagnostic yield of 60% including malignancy diagnosis). Major complications such as pneumothorax, pulmonary hemorrhage, and death occurred in 10% of patients. Complication rates were lower in the US-guided *vs* CT guided biopsy approach, which can be attributed to the fact that the biopsy of deeper parenchymal lesions was done only by CT guidance.

### Transbronchial Lung Biopsy

The utility of transbronchial lung biopsy (TBLB) is questionable secondary to the low diagnostic yield and increased risk of complications compared to bronchoscopy (49). In a large cohort of 130 adults post-HCT, transbronchial biopsy yield was <50% and <5% for infectious diagnostic yield. In addition, TBLB had a 3× higher risk of complications compared to BAL alone and did not lead to therapy modification. Transbronchial biopsies are performed less often in the pediatric HCT population as they render increased risk without a clear benefit.

## BAL Versus Lung Biopsy

Approximately 35% of pediatric HCT patients undergo BAL, and less often lung biopsy (8%) (34). Which procedure gives the most helpful diagnostic information with the lowest complication rate remains to be answered. Many studies have compared both procedures in immunocompromised children but found no consensus on the superiority of one procedure (30, 50). In a study that compared BAL to lung biopsy in a pediatric cohort post-HCT, the diagnostic yield of BAL was 40% compared to 94% with lung biopsy. The median days of intubation was 8.5 days in the BAL group compared to 4 days in the lung biopsy group (34). A meta-analysis of 95 studies in cancer and HCT patients (both adult and children) compared the efficacy and complications of BAL *vs* lung biopsy. Non-infectious etiology was diagnosed in lung biopsy more often than BAL; however, the rate of complication was higher in the lung biopsy group compared to the BAL group (0.15 *vs* 0.008) (51).

The decision of which procedure to choose depends on the situation and often requires a multidisciplinary team discussion among transplant, infectious, pulmonary, surgical, radiology, and ICU teams. If an infection or DAH is suspected, BAL is often the initial diagnostic approach. However, in some instances, even with the detection of a pathogen on BAL and giving appropriate treatment, respiratory status may not normalize. In these situations, multifactorial etiologies may be present and lung biopsy can likely establish other diagnosis. In addition, SLB should be the initial diagnostic tool when COP, BOS, pulmonary fibrosis, and/or treatment toxicity are suspected.

## Conclusion

Diagnosis of pulmonary complication post-HCT is complex and often requires a multidisciplinary approach. Initial diagnostic imaging may suggest the etiology of the pulmonary process. However, in many instances, further diagnostic testing such as BAL or lung biopsy is needed. BAL is useful for diagnosis of infectious pulmonary complications; however, lung biopsy is superior for diagnosis of non-infectious pulmonary complications. Benefits and risks of these procedures should be considered thoroughly, but an early decision aids in early identification and treatment of the pulmonary process and prevents further lung damage.

## Author Contributions

LE and AQ: conceptualization. LE, JM, CM, HA, MM, RR, SS, and AQ: writing—review and editing. All authors have reviewed and approved the final manuscript as submitted and agreed to be accountable for all aspects of the work. All authors contributed to the article and approved the submitted version.

## Funding

This research was funded by the American Lebanese Syrian Associated Charities (ALSAC).

## Conflict of Interest

The authors declare that the research was conducted in the absence of any commercial or financial relationships that could be construed as a potential conflict of interest.

## Publisher’s Note

All claims expressed in this article are solely those of the authors and do not necessarily represent those of their affiliated organizations, or those of the publisher, the editors and the reviewers. Any product that may be evaluated in this article, or claim that may be made by its manufacturer, is not guaranteed or endorsed by the publisher.

## References

[1] Elbahlawan L Srinivasan A Morrison RR. A Critical Care and Transplantation-Based Approach to Acute Respiratory Failure After Hematopoietic Stem Cell Transplantation in Children. Biol Blood Marrow Transplant (2016) 22:617–26. doi: 10.1016/j.bbmt.2015.09.015 PMC5033513 26409244

[2] Patriarca F Skert C Sperotto A Damiani D Cerno M Geromin A. Incidence, Outcome, and Risk Factors of Late-Onset Noninfectious Pulmonary Complications After Unrelated Donor Stem Cell Transplantation. Bone Marrow Transplant (2004) 33:751–8. doi: 10.1038/sj.bmt.1704426 14755316

[3] Elbahlawan L Galdo AM Ribeiro RC. Pulmonary Manifestations of Hematologic and Oncologic Diseases in Children. Pediatr Clin North Am (2021) 68:61–80. doi: 10.1016/j.pcl.2020.09.003 33228943

[4] Eikenberry M Bartakova H Defor T Haddad IY Ramsay NK Blazar BR. Natural History of Pulmonary Complications in Children After Bone Marrow Transplantation. Biol Blood Marrow Transplant (2005) 11:56–64. doi: 10.1016/j.bbmt.2004.09.008 15625545

[5] Park M Koh KN Kim BE Im HJ Seo JJ. Clinical Features of Late Onset Non-Infectious Pulmonary Complications Following Pediatric Allogeneic Hematopoietic Stem Cell Transplantation. Clin Transplant (2011) 25:E168–176. doi: 10.1111/j.1399-0012.2010.01357.x 21077955

[6] Lucena CM Torres A Rovira M Marcos MA de la Bellacasa JP Sánchez M. Pulmonary Complications in Hematopoietic SCT: A Prospective Study. Bone Marrow Transplant (2014) 49:1293–9. doi: 10.1038/bmt.2014.151 PMC7094728 25046219

[7] Elbahlawan L Rains KJ Stokes DC. Respiratory Care Considerations in the Childhood Cancer Patient. Respir Care (2017) 62:765–75. doi: 10.4187/respcare.05223 28546377

[8] Khosla J Yeh AC Spitzer TR Dey BR. Hematopoietic Stem Cell Transplant-Associated Thrombotic Microangiopathy: Current Paradigm and Novel Therapies. Bone Marrow Transplant (2018) 53:129–37. doi: 10.1038/bmt.2017.207 28967899

[9] Montani D Lau EM Dorfmüller P Girerd B Jaïs X Savale L. Pulmonary Veno-Occlusive Disease. Eur Respir J (2016) 47:1518–34. doi: 10.1183/13993003.00026-2016 27009171

[10] Harris B Geyer AI. Diagnostic Evaluation of Pulmonary Abnormalities in Patients With Hematologic Malignancies and Hematopoietic Cell Transplantation. Clin Chest Med (2017) 38:317–31. doi: 10.1016/j.ccm.2016.12.008 PMC7172342 28477642

[11] Barloon TJ Galvin JR Mori M Stanford W Gingrich RD. High-Resolution Ultrafast Chest CT in the Clinical Management of Febrile Bone Marrow Transplant Patients With Normal or Nonspecific Chest Roentgenograms. Chest (1991) 99:928–33. doi: 10.1378/chest.99.4.928 2009797

[12] Graham NJ Müller NL Miller RR Shepherd JD. Intrathoracic Complications Following Allogeneic Bone Marrow Transplantation: CT Findings. Radiology (1991) 181:153–6. doi: 10.1148/radiology.181.1.1887025 1887025

[13] Heussel CP Kauczor HU Heussel GE Fischer B Begrich M Mildenberger P. Pneumonia in Febrile Neutropenic Patients and in Bone Marrow and Blood Stem-Cell Transplant Recipients: Use of High-Resolution Computed Tomography. J Clin Oncol (1999) 17:796–805. doi: 10.1200/JCO.1999.17.3.796 10071269

[14] Korones DN Hussong MR Gullace MA. Routine Chest Radiography of Children With Cancer Hospitalized for Fever and Neutropenia: Is it Really Necessary? Cancer (1997) 80:1160–4. doi: 10.1002/(SICI)1097-0142(19970915)80:6<1160::AID-CNCR20>3.0.CO;2-5 9305718

[15] Maschmeyer G Donnelly JP. How to Manage Lung Infiltrates in Adults Suffering From Haematological Malignancies Outside Allogeneic Haematopoietic Stem Cell Transplantation. Br J Haematol (2016) 173:179–89. doi: 10.1111/bjh.13934 PMC7161791 26729577

[16] Roberts SD Wells GM Gandhi NM York NR Maron G Razzouk B. Diagnostic Value of Routine Chest Radiography in Febrile, Neutropenic Children for Early Detection of Pneumonia and Mould Infections. Support Care Cancer (2012) 20:2589–94. doi: 10.1007/s00520-011-1366-7 22278307

[17] Schueller G Matzek W Kalhs P Schaefer-Prokop C. Pulmonary Infections in the Late Period After Allogeneic Bone Marrow Transplantation: Chest Radiography Versus Computed Tomography. Eur J Radiol (2005) 53:489–94. doi: 10.1016/j.ejrad.2004.06.009 15741024

[18] Peña E Souza CA Escuissato DL Gomes MM Allan D Tay J. Noninfectious Pulmonary Complications After Hematopoietic Stem Cell Transplantation: Practical Approach to Imaging Diagnosis. Radiographics (2014) 34:663–83. doi: 10.1148/rg.343135080 24819788

[19] Kazerooni EA. High-Resolution CT of the Lungs. AJR Am J Roentgenol (2001) 177:501–19. doi: 10.2214/ajr.177.3.1770501 11517038

[20] Gottumukkala RV Kalra MK Tabari A Otrakji A Gee MS. Advanced CT Techniques for Decreasing Radiation Dose, Reducing Sedation Requirements, and Optimizing Image Quality in Children. Radiographics (2019) 39:709–26. doi: 10.1148/rg.2019180082 30924753

[21] Escuissato DL Gasparetto EL Marchiori E Rocha Gde M Inoue C Pasquini R. Pulmonary Infections After Bone Marrow Transplantation: High-Resolution CT Findings in 111 Patients. AJR Am J Roentgenol (2005) 185:608–15. doi: 10.2214/ajr.185.3.01850608 16120907

[22] Franquet T Rodriguez S Martino R Giménez A Salinas T Hidalgo A. Thin-Section CT Findings in Hematopoietic Stem Cell Transplantation Recipients With Respiratory Virus Pneumonia. AJR Am J Roentgenol (2006) 187:1085–90. doi: 10.2214/AJR.05.0439 16985161

[23] Alexander BD Lamoth F Heussel CP Prokop CS Desai SR Morrissey CO. Guidance on Imaging for Invasive Pulmonary Aspergillosis and Mucormycosis: From the Imaging Working Group for the Revision and Update of the Consensus Definitions of Fungal Disease From the EORTC/MSGERC. Clin Infect Dis (2021) 72:S79–s88. doi: 10.1093/cid/ciaa1855 33709131

[24] Donnelly JP Chen SC Kauffman CA Steinbach WJ Baddley JW Verweij PE. Revision and Update of the Consensus Definitions of Invasive Fungal Disease From the European Organization for Research and Treatment of Cancer and the Mycoses Study Group Education and Research Consortium. Clin Infect Dis (2020) 71:1367–76. doi: 10.1093/cid/ciz1008 PMC7486838 31802125

[25] Chong S Kim TS Cho EY. Herpes Simplex Virus Pneumonia: High-Resolution CT Findings. Br J Radiol (2010) 83:585–9. doi: 10.1259/bjr/51409455 PMC3473669 20442279

[26] Song I Yi CA Han J Kim DH Lee KS Kim TS. CT Findings of Late-Onset Noninfectious Pulmonary Complications in Patients With Pathologically Proven Graft-Versus-Host Disease After Allogeneic Stem Cell Transplant. AJR Am J Roentgenol (2012) 199:581–7. doi: 10.2214/AJR.11.7165 22915397

[27] Franquet T Müller NL Lee KS Giménez A Flint JD. High-Resolution CT and Pathologic Findings of Noninfectious Pulmonary Complications After Hematopoietic Stem Cell Transplantation. AJR Am J Roentgenol (2005) 184:629–37. doi: 10.2214/ajr.184.2.01840629 15671389

[28] Ooi GC Peh WC Ip M. High-Resolution Computed Tomography of Bronchiolitis Obliterans Syndrome After Bone Marrow Transplantation. Respiration (1998) 65:187–91. doi: 10.1159/000029257 9670299

[29] Ben-Ari J Yaniv I Nahum E Stein J Samra Z Schonfeld T. Yield of Bronchoalveolar Lavage in Ventilated and Non-Ventilated Children After Bone Marrow Transplantation. Bone Marrow Transplant (2001) 27:191–4. doi: 10.1038/sj.bmt.1702773 11281389

[30] Armenian SH Hoffman JA Butturini AM Kapoor N Mascarenhas L. Invasive Diagnostic Procedures for Pulmonary Infiltrates in Pediatric Hematopoietic Stem Cell Transplant Recipients. Pediatr Transplant (2007) 11:736–42. doi: 10.1111/j.1399-3046.2007.00733.x 17910650

[31] Kasow KA King E Rochester R Tong X Srivastava DK Horwitz EM. Diagnostic Yield of Bronchoalveolar Lavage is Low in Allogeneic Hematopoietic Stem Cell Recipients Receiving Immunosuppressive Therapy or With Acute Graft-Versus-Host Disease: The St. Jude Experience, 1990-2002. Biol Blood Marrow Transplant (2007) 13:831–7. doi: 10.1016/j.bbmt.2007.03.008 17580261

[32] Efrati O Gonik U Bielorai B Modan-Moses D Neumann Y Szeinberg A. Fiberoptic Bronchoscopy and Bronchoalveolar Lavage for the Evaluation of Pulmonary Disease in Children With Primary Immunodeficiency and Cancer. Pediatr Blood Cancer (2007) 48:324–9. doi: 10.1002/pbc.20784 16568442

[33] Forslöw U Remberger M Nordlander A Mattsson J. The Clinical Importance of Bronchoalveolar Lavage in Allogeneic SCT Patients With Pneumonia. Bone Marrow Transplant (2010) 45:945–50. doi: 10.1038/bmt.2009.268 19784077

[34] Qualter E Satwani P Ricci A Jin Z Geyer MB Alobeid B. A Comparison of Bronchoalveolar Lavage Versus Lung Biopsy in Pediatric Recipients After Stem Cell Transplantation. Biol Blood Marrow Transplant (2014) 20:1229–37. doi: 10.1016/j.bbmt.2014.04.019 PMC4099252 24769329

[35] Nadimpalli S Foca M Satwani P Sulis ML Constantinescu A Saiman L. Diagnostic Yield of Bronchoalveolar Lavage in Immunocompromised Children With Malignant and Non-Malignant Disorders. Pediatr Pulmonol (2017) 52:820–6. doi: 10.1002/ppul.23644 PMC7167680 28052585

[36] Tang FF Zhao XS Xu LP Zhang XH Chen YH Mo XD. Utility of Flexible Bronchoscopy With Polymerase Chain Reaction in the Diagnosis and Management of Pulmonary Infiltrates in Allogeneic HSCT Patients. Clin Transplant (2018) 32(1):e13146. doi: 10.1111/ctr.13146 PMC7162290 29090481

[37] Eroglu-Ertugrul NG Yalcin E Oguz B Ocal T Kuskonmaz B Emiralioglu N. The Value of Flexible Bronchoscopy in Pulmonary Infections of Immunosuppressed Children. Clin Respir J (2020) 14:78–84. doi: 10.1111/crj.13103 31710418 PMC7162225

[38] Shannon VR Andersson BS Lei X Champlin RE Kontoyiannis DP. Utility of Early Versus Late Fiberoptic Bronchoscopy in the Evaluation of New Pulmonary Infiltrates Following Hematopoietic Stem Cell Transplantation. Bone Marrow Transplant (2010) 45:647–55. doi: 10.1038/bmt.2009.203 19684637

[39] Huang J Jiang E Yang D Wei J Zhao M Feng J. Metagenomic Next-Generation Sequencing Versus Traditional Pathogen Detection in the Diagnosis of Peripheral Pulmonary Infectious Lesions. Infect Drug Resist (2020) 13:567–76. doi: 10.2147/IDR.S235182 PMC7036976 32110067

[40] Dunn JC West KW Rescorla FJ Tres Scherer LR Engum SA Rouse TM. The Utility of Lung Biopsy in Recipients of Stem Cell Transplantation. J Pediatr Surg (2001) 36:1302–3. doi: 10.1053/jpsu.2001.25799 11479881

[41] Shorter NA Ross AJ 3rd August C Schnaufer L Zeigler M Templeton JM. The Usefulness of Open-Lung Biopsy in the Pediatric Bone Marrow Transplant Population. J Pediatr Surg (1988) 23:533–7. doi: 10.1016/S0022-3468(88)80363-4 3047358

[42] Hayes-Jordan A Benaim E Richardson S Joglar J Srivastava DK Bowman L. Open Lung Biopsy in Pediatric Bone Marrow Transplant Patients. J Pediatr Surg (2002) 37:446–52. doi: 10.1053/jpsu.2002.30854 11877664

[43] Wang JY Chang YL Lee LN Chen JH Tang JL Yang PC. Diffuse Pulmonary Infiltrates After Bone Marrow Transplantation: The Role of Open Lung Biopsy. Ann Thorac Surg (2004) 78:267–72. doi: 10.1016/j.athoracsur.2004.03.002 15223441

[44] Gassas A Craig-Barnes H Dell SD Cox P Schechter T Doyle J. Severe Lung Injury and Lung Biopsy in Children Post-Hematopoietic Stem Cell Transplantation: The Differences Between Allogeneic and Autologous Transplantation. Pediatr Transplant (2013) 17:278–84. doi: 10.1111/petr.12060 23461864

[45] Uhlving HH Andersen CB Christensen IJ Gormsen M Pedersen KD Buchvald F. Biopsy-Verified Bronchiolitis Obliterans and Other Noninfectious Lung Pathologies After Allogeneic Hematopoietic Stem Cell Transplantation. Biol Blood Marrow Transplant (2015) 21:531–8. doi: 10.1016/j.bbmt.2014.12.004 25498923

[46] Ortega-Laureano L Santiago T Maron G Davidoff AM Fernandez-Pineda I. Surgical Lung Biopsy in Children After Hematopoietic Cell Transplantation. J Pediatr Surg (2018) 53:1129–33. doi: 10.1016/j.jpedsurg.2018.02.069 29602553

[47] Dieffenbach BV Madenci AL Murphy AJ Weldon CB Weil BR Lehmann LE. Therapeutic Impact and Complications Associated With Surgical Lung Biopsy After Allogeneic Hematopoietic Stem Cell Transplantation in Children. Biol Blood Marrow Transplant (2019) 25:2181–5. doi: 10.1016/j.bbmt.2019.06.026 31255742

[48] Cleveland H Chau A Jeng Z Gardner G Yoo R Zhang W. Percutaneous Lung Biopsy in Immunocompromised Pediatric Patients. J Vasc Interv Radiol (2020) 31:93–8. doi: 10.1016/j.jvir.2019.07.016 31767410

[49] O’Dwyer DN Duvall AS Xia M Hoffman TC Bloye KS Bulte KS. Transbronchial Biopsy in the Management of Pulmonary Complications of Hematopoietic Stem Cell Transplantation. Bone Marrow Transplant (2018) 53:193–8. doi: 10.1038/bmt.2017.238 PMC5803310 29058699

[50] Elbahlawan LM Avent Y Montoya L Wilder K Pei D Cheng C. Safety and Benefits of Bronchoalveolar Lavage and Lung Biopsy in the Management of Pulmonary Infiltrates in Children With Leukemia. J Pediatr Hematol Oncol (2016) 38:597–601. doi: 10.1097/MPH.0000000000000644 27467366 PMC5699503

[51] Chellapandian D Lehrnbecher T Phillips B Fisher BT Zaoutis TE Steinbach WJ. Bronchoalveolar Lavage and Lung Biopsy in Patients With Cancer and Hematopoietic Stem-Cell Transplantation Recipients: A Systematic Review and Meta-Analysis. J Clin Oncol (2015) 33:501–9. doi: 10.1200/JCO.2014.58.0480 25559816

